# Genetics, genomics and breeding of groundnut (*Arachis hypogaea* L.)

**DOI:** 10.1111/pbr.12645

**Published:** 2018-08-29

**Authors:** Haile Desmae, Pasupuleti Janila, Patrick Okori, Manish K. Pandey, Babu N. Motagi, Emmanuel Monyo, Omari Mponda, David Okello, Dramane Sako, Candidus Echeckwu, Richard Oteng‐Frimpong, Amos Miningou, Chris Ojiewo, Rajeev K. Varshney

**Affiliations:** ^1^ International Crop Research Institute for the Semi‐Arid Tropics (ICRISAT) Bamako Mali; ^2^ ICRISAT Patancheru India; ^3^ ICRISAT Lilongwe Malawi; ^4^ ICRISAT Kano Nigeria; ^5^ ICRISAT Nairobi Kenya; ^6^ Division of Research and Development (DRD) Tanzania Agricultural Research Institute (TARI) ‐ Naliendele Mtwara Tanzania; ^7^ National Agricultural Research Organization (NARO) Entebbe Uganda; ^8^ Institut d'Economie Rurale (IER) Bamako Mali; ^9^ Institute of Agricultural Research (IAR) Zaria Nigeria; ^10^ Savanah Agricultural Research Institute (SARI) Tamale Ghana; ^11^ Institut National d'Environnement et de Recherches Agricoles (INERA) Ouagadougou Burkina Faso

**Keywords:** breeding, genetic maps, genetics, genomics, groundnut, molecular markers, QTL

## Abstract

Groundnut is an important food and oil crop in the semiarid tropics, contributing to household food consumption and cash income. In Asia and Africa, yields are low attributed to various production constraints. This review paper highlights advances in genetics, genomics and breeding to improve the productivity of groundnut. Genetic studies concerning inheritance, genetic variability and heritability, combining ability and trait correlations have provided a better understanding of the crop's genetics to develop appropriate breeding strategies for target traits. Several improved lines and sources of variability have been identified or developed for various economically important traits through conventional breeding. Significant advances have also been made in groundnut genomics including genome sequencing, marker development and genetic and trait mapping. These advances have led to a better understanding of the groundnut genome, discovery of genes/variants for traits of interest and integration of marker‐assisted breeding for selected traits. The integration of genomic tools into the breeding process accompanied with increased precision of yield trialing and phenotyping will increase the efficiency and enhance the genetic gain for release of improved groundnut varieties.

## 1 INTRODUCTION

Groundnut (*Arachis hypogaea* L.), also known as peanut, is a member of genus *Arachis* and family *Leguminosae* (Krapovickas & Gregory, 1994). It is believed to have originated in the southern Bolivia to northern Argentina region of South America. The genus *Arachis* contains 80 species, and most of them are diploid (2*n* = 2x = 20) with only two allotetraploids. The cultivated groundnut is allotetraploid (AABB, 2*n* = 4x = 40), which is believed to be the result of hybridization between two wild species, *Arachis duranensis* (AA‐genome, 2*n* = 2x = 20) named as “A‐genome ancestor” and *Arachis ipaensis* (BB‐genome, 2*n* = 2x = 20) named as “B‐genome ancestor” and subsequent chromosome doubling. Based on the patterns of reproductive and vegetative branching and on the pod morphology, the cultivated species is divided into two cultivated subspecies that is *A. hypogaea* subsp. *hypogaea* and *A. hypogaea* subsp. *fastigiata*. The subspecies are further divided into botanical varieties. The subsp. *hypogaea* is divided into *hypogaea* (virginia) and hirsuta, while the subsp. *fastigiata* is divided into *fastigiata* (valencia), *vulgaris* (Spanish), *peruviana* and *aequatoriana* (Krapovickas & Gregory, 1994).

Groundnut is grown in more than 100 countries covering over 26 million (M) hectares (ha) area in 2014 with a global production of about 44 M metric tons and an average yield of about 1,655 kg/ha (FAOSTAT 2017). Asia (58.3%) and Africa (31.6%) accounted for about 90% of the world's production with China (16.6 M tons), India (6.6 M tons) and Nigeria (3.4 M tons) being the top three largest producing countries (FAOSTAT, 2017). The groundnut seed contains 22% to 30% protein and 35% to 60% oil and is a rich source of dietary fibre, minerals, vitamins and bioactive compounds, hence contributing to household nutrition. It is suitable for making nutrient‐dense foods for alleviating malnutrition in vulnerable groups such as pregnant and breastfeeding women and children under 2 years, particularly in developing countries (Anim‐Somuah, Henson, Humphrey, & Robinson, 2013). The haulms and groundnut cake are important sources of animal feed. In addition, groundnut has the ability to fix atmospheric nitrogen benefitting the succeeding crop. As a cash crop, it is frequently traded locally, regionally and globally, significantly contributing to rural household cash income and national economy. In the west and central Africa (WCA), for example, groundnut accounts for up to 50% or more of rural household cash income in many countries—46% in Mali, 54% in Nigeria, 66% in Niger and 80% in Senegal (GAIN 2010; Ndjeunga et al., 2010). In Asia and Africa, a large number of women and youth are engaged in the cultivation, processing and marketing of groundnut, thereby contributing to their economic participation and empowerment. In Nigeria, for example, almost all the small‐scale groundnut oil processing is controlled by women. In Mali, about 85% of groundnut fields are owned by women (Ndjeunga et al., 2010).

Groundnut productivity significantly varies among regions with Africa having the lowest mean yield of around 965 kg/ha (FAOSTAT 2017). In Asia, the productivity is relatively better with an average yield of 2,370 kg/ha. On the other hand, in the USA and other developed countries, groundnut yields are high with a yield over 3,300 kg/ha. In general, groundnut productivity has significantly increased over the last five decades with a global yield average increasing from 849 kg/ha in 1961 to 1655 kg/ha in 2014, which is attributed to significant advances in genetics, genomics, breeding and crop management. This paper reviews the advances in understanding the genetics of important traits, genome sequences, molecular marker development, QTL analysis, genetic resources, breeding for specific traits and integration of genomic tools into groundnut breeding process to enhance the genetic gain and improve the productivity of the crop.

## 2 GENETICS

Detailed reviews on groundnut genetics covering inheritance, cytogenetics, combining ability, genotypic and phenotypic coefficients of variation, heritability, genetic gain, genotype‐by‐environment interactions and trait correlations were published (Knauft & Wynne, 1995; Nigam, 2014; Reddy, 1988). Qualitative and quantitative inheritances of traits have been reported. Generally, majority of morphological (e.g., growth and branching, leaf, pod and seed traits), quality (e.g., protein and oil) and disease resistance (leaf spots, rust) traits were reported to have predominantly qualitative inheritance (e.g., Asibuo et al., 2008; Gangadhara & Nadaf, 2016; Jakkeral, Nadaf, Gowda, & Bhat, 2013; Pattanashetti, Gowda, & Girija, 2008; Upadhyaya & Nigam, 1994, 1998, 1999). But quantitative inheritances were also reported for some of the traits such as oil content and quality (Aruna & Nigam, 2009; Dwivedi, Pande, Rao, & Nigam, 2002; Khedikar et al., 2010; Pandey, Wang, et al., 2014; Sarvamangala, Gowda, & Varshney, 2011; Shasidhar et al., 2017; Sujay et al., 2012; Wilson et al., 2017). Most of the economically important traits such as yield, maturity and drought tolerance traits are quantitatively inherited (Knauft & Wynne, 1995; Nageswara Rao, Talwar, & Wright, 2001; Ravi et al., 2011; Upadhyaya, 2005; Upadhyaya & Nigam, 1998). The presence of genetic and nongenetic variances was reported for various traits (Dwivedi, Nigam, Chandra, & Ramraj, 1998; Janila, Ramaiah, et al., 2013; John, Reddy, Reddy, Sudhakar, & Reddy, 2011; Pattanashetti et al., 2008; Upadhyaya, Gopal, Nadaf, & Vijayakumar, 1992).

Low‐to‐high genotypic and phenotypic coefficients of variation, broad‐sense heritability, genetic advance and genetic advance as percentage of mean were reported for various traits including grain and pod yield, days to 50% flowering and plant height, shelling percentage, specific leaf area (SLA) and SPAD chlorophyll meter readings (SCMR), number of pods per plant and 100‐seed weight (e.g., John, Vasanthi, Sireesha, & Krishna, 2013; John et al., 2011; Padmaja, Eswari, BrahmeswaraRao, & Madhusudhan Reddy, 2013; Padmaja, Eswari, BrahmeswaraRao, & Prasad, 2015; Patil, Punewar, Nandanwar, & Shah, 2014; Songsri et al., 2009; Thirumala Rao, Venkanna, Bhadru, & Bharathi, 2014; Upadhyaya, 2005). In the case of trait correlations, grain and pod yield were reported to be positively correlated among themselves and with traits such as shelling percentage, biomass production, 100‐seed weight, number of pods per plant and dry haulm yield (e.g., Padmaja et al., 2013, 2015; Thirumala Rao et al., 2014) and also with drought‐related traits such as harvest index (HI), SCMR and SLA (e.g., Songsri et al., 2009; Upadhyaya, Sharma, Singh, & Singh, 2011). On the other hand, negative correlations were reported for grain and pod yield with early leaf spot (ELS) resistance parameters, days to first flowering and days to 50% flowering (Gaikpa, Akromah, Asibuo, Appiah‐Kubi, & Nyadanu, 2015; Padmaja et al., 2013). For quality traits, negative correlations between protein content and oil content and between oleic acid and linoleic acid were reported (Sarvamangala et al., 2011).

## 3 GENOMICS

Limited genomic resources existed for groundnut prior to 2005 (Pandey et al., 2012). However, significant advances have been made in recent years in genome sequencing, development of molecular markers, construction of genetic maps and quantitative trait locus (QTL) analyses. Various marker systems including RFLP (restriction fragment length polymorphism), RAPD (random amplification of polymorphic DNA), AFLP (amplified fragment length polymorphism), DArT (diversity array technology), SSR (simple sequence repeat) and SNPs (single‐nucleotide polymorphisms) were developed (Pandey et al., 2012; Varshney, 2016) and have been utilized for genetic diversity analyses, constructing genetic maps, mapping of traits of breeding interest and marker‐assisted breeding. The emphasis has been more on SSR and SNP markers for usefulness and practical reasons. SSR markers are codominant, more informative and easy to score in the tetraploid genome, while SNP markers are highly amenable to high‐throughput genotyping approaches (Bertioli et al., 2014; Pandey et al., 2012). Consequently, a large number of expressed sequence tag (EST)‐based SSR markers ranging from 26 (Hopkins et al., 1999) to 6455 (Peng, Gallo, Tillman, Rowland, & Wang, 2016) have been reported. Similarly, large numbers of SNP markers have been developed including 8486 candidate SNPs from a screening of sequences of 17 genotypes assembled along with sequences from the reference ‘Tifrunner’ transcriptome (Alves et al., 2008; GCP 2011), which was used to construct 1536‐SNP GoldenGate assay (Nagy et al., 2012). Another 768‐SNP Illumina GoldenGate assay was developed at the University of California‐Davis (Pandey et al., 2012). These assays were found very informative for genotyping diploid species, but limited use for tetraploid species (Bertioli et al., 2014; Pandey et al., 2012). Zhou et al. (2014) reported the development of 53,257 SNPs for tetraploid species. Additional SNPs have become available including 62 SNPs (Hong et al., 2015), 263,840 SNPs and indel variants (Chopra et al., 2015), 11,902 SNPs (Peng et al., 2016) and 6965 SNPs (Peng et al., 2017). Besides, 96 SNP markers were converted to kompetitive allele‐specific PCR (KASPar) SNP markers to develop KASPar assays designated as GKAMs (groundnut KASPar assay markers) for use in LGC's KASP genotyping service (Khera et al., 2013). Similarly, easy‐to‐use KASP markers linked to root‐knot nematode (RKN) resistance loci were developed and validated in a tetraploid context (Leal‐Bertioli et al., 2015).

Genetic maps were constructed to understand the groundnut genome structure and organization and to identify QTLs for traits of breeding interest. Different marker systems such as RFLP (Halward, Stalker, & Kochert, 1993), RAPD (Garcia, Stalker, Schroeder, Lyerly, & Kocher, 2005), AFLP (Herselman, Thwaites, Kimmins, & Seal, 2004), SSR (Moretzsohn, Barbosa, Alves‐Freitas, Teixeira, & Leal‐Bertioli, 2009), SNP (Bertioli et al., 2014) and DArT (Shasidhar et al., 2017) were employed to construct the genetic maps, but the majority of maps were based on SSR markers from biparental populations (Table 1). Earlier SSR‐based genetic maps had lower marker density (e.g., 135 markers, Varshney et al., 2009), but as more and more SSR markers have become available, the genetic maps were improved with more dense maps developed recently (e.g., 1,469 markers—Shirasawa et al., 2013). SNP and other markers were integrated into some of the genetic maps. Besides, six consensus maps were developed, the first with 175 loci (Hong et al., 2010) and the latest with 3,693 loci (Shirasawa et al., 2013), which are useful for the characterization of the groundnut genome. Specifically, the construction of the consensus map by Shirasawa et al. (2013) from 16 segregating populations of diverse genetic backgrounds has enabled mapping a larger number of loci with greater genome coverage than in any of the genetic maps from the single populations and was useful to determine the relative position of common markers across different mapping populations. While many genetic maps were developed with a focus on mapping maximum number of loci onto a single map (e.g., Foncéka et al., 2009; Hong et al., 2008, 2010; Shirasawa et al., 2013; Wang et al., 2012), majority of them were developed with a focus on facilitating QTL analysis (trait mapping) and development of diagnostic markers for marker‐assisted breeding. QTL analysis studies to date have reported the identification of more than 1,380 small and major effect QTLs (Table 2) for various traits including agronomic and yield component traits (e.g., Luo, Xu, et al., 2017; Selvaraj et al., 2009), quality traits (e.g., Sarvamangala et al., 2011; Shasidhar et al., 2017), biotic stress resistance (e.g., Khedikar et al., 2010; Kolekar et al., 2016; Pandey, Wang, et al., 2017; Pandey, Khan, et al., 2017; Zhou et al., 2016) and abiotic stress resistance mainly for drought‐related traits (e.g., Leal‐Bertioli et al., 2016; Varshney et al., 2009).

**Table 1:** Genetic maps for diploid and tetraploid *Arachis* species

Genome	Population	Population size	Marker loci mapped	Marker type	LGs	Total map distance (cM)	References
AA	*A. stenosperma* × *A. cardenasii*	87 F2	132	RFLP	11	1,063.00	Halward et al., 1993;
	[*A. stenosperma* × (*A. stenosperma* × *A. cardenasii*)]	44 BC1F1	206	RAPD, RFLP	11	800	Garcia et al., 2005;
	*A. duranensis* (K7988) × *A. stenosperma* (V10309)	93 F2	204	SSR	11	1,230.89	Moretzsohn et al., 2005;
		93 F2	369	SSR, anchor, AFLP, NBS profiling, SNP, RGA‐RFLP SCAR	10	–	Leal‐Bertioli et al., 2009;
		89 F5	597	SSR, TE	10	544.00	Shirasawa et al., 2013;
		90 F5	384	SNP, SSR	10	705.10	Bertioli et al., 2014;
		93 F6	502	SNP, SSR, RGA, anchor, morphological	10	1,004.10	Leal‐Bertioli et al., 2016;
	*A. duranensis* (PI 475887) × *A. duranensis* (Grif 15036)	94 F2	1,724	SNP, SSR, SSCP, RGC	10	1,081.30	Nagy et al., 2012;
BB	*A. ipaënsis* (K30076) × *A. magna* (K30097)	93 F2	149	SSR	10	1,294.00	Moretzsohn et al., 2009;
		94 RILs	798	SSR, TE	10	461.00	Shirasawa et al., 2013;
		94 RILs	399	SSR, TE	10	678.00	Leal‐Bertioli et al., 2015;
	K 9484 (PI 298639) × GKBSPSc 30081 (PI 468327) in *A. batizocoi*	94 F2	449	SSR	16	1,278.60	Guo et al., 2012;
AABB	Florunner × TxAG‐6 {[*A. batizocoi* K9484 × (*A. cardenasii* GKP10017 × *A. diogoi* GKP10602)]^4×^}	78 BC1F1	370	RFLP	23	2,210.00	Burow, Simpson, Starr, & Paterson, 2001;
		78 BC1F1	91	SSR	22	1,321.90	Wilson et al., 2017;
	ICG 12991 × ICGV‐SM 93541	60 F2	12	AFLP	5	139.4	Herselman et al., 2004;
	[Fleur 11 × (*A. ipaënsis* × *A. duranensis*)^4×^]	88 BC1F1	298	SSR	21	1,843.70	Foncéka et al., 2009;
	Yueyou 13 × Zhenzhuhei	142 RILs	131	SSR	20	679.00	Hong et al., 2008;
	TAG 24 × ICGV 86031	318 RILs	135	SSR	22	1,270.50	Varshney et al., 2009;
		318 RILs	191	SSR	22	1,785.40	Ravi et al., 2011;
	Yueyou 13 × Zhenzhuhei	142 F4:6	132	SSR	19	684.90	Hong et al., 2010;
	Yueyou 13 × Fu 95‐5	84 F4:6	109	SSR	21	540.69	Hong et al., 2010;
	Yueyou 13 × J11	136 F4:6	46	SSR	13	401.70	Hong et al., 2010;
	TAG 24 × GPBD 4	268 RILs	56	SSR	14	462.24	Khedikar et al., 2010;
		266 RILs	188	SSR	20	1,922.40	Sujay et al., 2012;
		266 RILs	289	SSR, TE	20	1,730.80	Kolekar et al., 2016;
	TG 26 × GPBD 4	146 RILs	45	SSR	8	657.90	Sarvamangala et al., 2011;
		146 RILs	181	SSR	21	1,963.00	Sujay et al., 2012;
	ICGS 44 × ICGS 76	188 RILs	82	SSR	15	831.40	Gautami, Pandey, et al., 2012;
	ICGS 76 × CSMG 84‐1	177 RILs	119	SSR	20	2,208.20	Gautami, Pandey, et al., 2012;
	SunOleic 97R × NC94022	352 RILs	172	SSR, CAPs	22	920.70	Qin et al., 2012;
		352 RILs	206	SSR, CAPs	20	1,780.60	Pandey, Wang, et al., 2014;
		352 RILs	248	SSR	21	1,425.90	Khera et al., 2016;
	Tifrunner × GT‐C20	94 F2	318	SSR	21	1,674.40	Wang et al., 2012;
		248 RILs	239	SSR, CAPs	26	1,213.40	Qin et al., 2012;
		248 RILs	378	SSR, CAPs	20	2,487.40	Pandey, Wang, et al., 2014;
		248 RILs	418	SSR	20	1,935.40	Pandey, Wang, et al., 2017;
	YI‐0311 × Nakateyutaka	186 F2	326	SSR, TE	19	1,332.90	Shirasawa et al., 2012;
	Satonoka × Kintoki	94 F2	1,114	SSR, TE	21	2,166.40	Shirasawa et al., 2012;
	*A. hypogaea* “Runner IAC 886” × (*A. ipaensis* × *A. duranensis*)^4x^	91 RILs	1,469	SSR, TE	20	1,442.00	Shirasawa et al., 2013;
		89 F6	772	SNP, SSR	20	1,487.30	Bertioli et al., 2014;
	Zhonghua 5 × ICGV 86699	166 RILs	1,685	SNP, SSR	20	1,446.70	Zhou et al., 2014;
	VG 9514 × TAG 24	164 RILS	95	SSR	24	882.90	Mondal et al., 2012;
		164 RILs	190	SSR, ISSR, TE, RGC	21	1,796.70	Mondal, Hadapad, Hande, & Badigannavar, 2014;
	Zhonghua 10 × ICG12625	232 F2	470	SSR	20	1,877.30	Huang et al., 2015;
		140 RILs	1,219	SSR, TE	20	2,038.75	Huang et al., 2016;
	Fuchuan Dahuasheng × ICG 6375	218 F2:3	347	SSR	22	1,675.60	Chen, Jiao, et al., 2016;
	Xuhua 13 × Zhonghua 6	282 F2:3	228	SSR	22	1,337.70	Chen, Jiao, et al., 2016;
	Florida‐ EP™ “113” × Georgia Valencia	163 F2	30	SSR, SNP	1	157.80	Tseng et al., 2016;
	ICGV 00350 × ICGV 97045	268 F2	1,152	DArT, DArTseq	20	2,423.12	Vishwakarma et al., 2016;
	79266 × D893	151 RILs	231	SSR	23	905.18	Li et al., 2017;
	Yuanza 9102 × Xuzhou 68‐4	195 RILs	743	SSR	22	1,232.57	Luo, Ren, et al., 2017;
		195 RILS	830	SSR	20	1,386.19	Luo, Xu, et al., 2017;
	ICGV 07368 × ICGV 06420	184 F2	854	DArT, SSR	20	3,526.00	Shasidhar et al., 2017;
	ICGV 06420 × SunOleic 95R	179 F2	1,435	DArT, DArTseq	20	1,869.00	Shasidhar et al., 2017;
	Tamrun OL07 × Tx964117	90 RILs	1,211	SNP	20	–	Liang, Baring, Wang, & Septiningsih, 2017;
	TMV 2 × TMV 2‐NLM	432 RILS	91	TE	20	1,205.66	Hake et al., 2017;
Consensus	3 populations	–	175	SSR	22	885.40	Hong et al., 2010;
	2 populations	–	225	SSR	20	1,152.90	Sujay et al. 2012
	3 populations	–	293	SSR	20	2,840.80	Gautami, Pandey, et al., 2012;
	2 populations	–	324	SSR	21	1,352.10	Qin et al., 2012;
	11 populations	–	897	SSR	20	3,863.60	Gautami, Foncéka, et al., 2012;
	16 populations	–	3,693	SSR, TE	20	2,651.00	Shirasawa et al., 2013

Note

TE: transposon elements; RGC: resistance gene candidate; RGA: resistance gene analogue.

**Table 2:** Reported QTLs for important traits of breeding interest in groundnut

Trait group	Trait	Number of QTLs identified			Population	Reference
		Total^a^	PVE	Major		
Agronomic and yield component traits	GH, plant spread, MSH, PH, total biomass, DF, PoM, LNB, haulm weight, shell weight, shelling %, HI, pod number, pod weight, seed number,100‐SW, pod beak, pod constriction, pod length, pod width, seed width, seed length, FSD	7	9.19–17.69	5	Tamrun OL01 × BSS 56	Selvaraj et al., 2009;
		106	8.50–26.70	29	[Fleur 11 × (*A. ipaënsis* × *A. duranensis*)^4×^]	Foncéka, Tossim, Rivallan, Vignes, Faye, et al., 2012; Foncéka, Tossim, Rivallan, Vignes, Lacut, et al., 2012;
		23	4.80–28.20	17	Satonoka × Kintoki	Shirasawa et al., 2012;
		25	6.20–30.40	9	*A. ipaënsis* (K30076) × *A. magna* (K30097)	Leal‐Bertioli et al., 2015;
		31	8.30–26.00	263	*A. ipaënsis* (K30076) × *A. magna* (K30097)	Leal‐Bertioli et al., 2016;
		24	1.69–18.70	11	Zhonghua 10 × ICG 12625	Huang et al., 2015;
		18	4.85–20.52	8	Zhonghua 10 × ICG 12625	Huang et al., 2016;
		22	2.55–7.95	0	Zhonghua 5 × ICGV 86699	Zhou et al., 2016;
		39	1.25–26.11	13	Fuchuan Dahuasheng × ICG 6375; Xuhua 13 × Zhonghua 6	Chen, Jiao, et al., 2016;
		2	22.14–71.21	2	ICGV 00350 × ICGV 97045	Vishwakarma et al., 2016;
		7	6.12–22.53	2	79266 × D893	Li et al., 2017;
		25	4.46–17.01	5	Yuanza 9102 × Xuzhou 68‐4	Luo, Ren, et al., 2017;
		42	3.68–27.84	11	Yuanza 9102 × Xuzhou 68‐4	Luo, Xu, et al., 2017;
		86	3.84–15.06	6	TAG 24 × GPBD 4	Khedikar et al., 2018;
		‐	12.00–32.30	6	TMV 2 × TMV 2‐NLM	Hake et al., 2017;
Quality traits	Linoleic acid, oleic acid, O/L ratio and other fatty acids	3	5.10–9.70	0	TG 26 × GPBD 4	Sarvamangala et al., 2011;
		27	1.04–42.33	17	SunOleic 97R × NC94022, Tifrunner × GT‐C20	Pandey, Wang, et al., 2014;
		191	0.16–40.56	34	SunOleic 97R × NC94022, Tifrunner × GT‐C20	Wang et al., 2015;
		11	1.72–20.20	7	Zhonghua 10 × ICG 12625	Huang et al., 2015;
		48	2.00–17.00	5	Florunner × TxAG‐6	Wilson et al., 2017;
		21	8.40–78.60	20	ICGV 06420 × SunOleic 95R	Shasidhar et al., 2017;
		–	15.10	1	TMV 2 × TMV 2‐NLM	Hake et al., 2017;
	Oil content	1	11.03	1	Tamrun OL01 × BSS 56	Selvaraj et al., 2009;
		4	1.50–9.10	0	TG 26 × GPBD 4	Sarvamangala et al., 2011;
		15	2.53–10.23	5	SunOleic 97R × NC94022; Tifrunner × GT‐C20	Pandey, Wang, et al., 2014;
		1	14.36	1	Zhonghua 10 × ICG 12625	Huang et al., 2015;
		13	2.00–18.00	2	Florunner × TxAG‐6	Wilson et al., 2017;
		8	5.60–22.10	2	ICGV 07368 × ICGV 06420	Shasidhar et al., 2017;
	Protein content	6	1.50–10.70	2	TG 26 × GPBD 4	Sarvamangala et al., 2011;
		–	26.40	1	TMV 2 × TMV 2‐NLM	Hake et al., 2017;
Resistance to abiotic stress	T, TE, SLA, LA, SCMR, CI, CC, yield components measured under drought stress	38	2.90–17.60	6	TAG 24 × ICGV 86031	Varshney et al., 2009;
		105	3.28–33.36	–	TAG 24 × ICGV 86031	Ravi et al., 2011;
		178	1.70–40.10	0	ICGS 76 × CSMG 84‐1; ICGS 44 × ICGS 76	Gautami, Pandey, et al., 2012;
		12	8.50–31.20	8	*A. ipaënsis* (K30076) × *A. magna* (K30097)	Leal‐Bertioli et al., 2016;
		13	10.40–20.10	13	[Fleur 11 × (*A. ipaënsis* × *A. duranensis*)^4×^]	Foncéka et al., 2012;
Resistance to biotic stress	Rust resistance	12	1.70–55.20	1	TAG 24 × GPBD 4	Khedikar et al., 2010;
		15	2.54–82.96	7	TAG 24 × GPBD 4; TG 26 × GPBD 4	Sujay et al., 2012;
		13	5.80–59.30	2	*A. ipaënsis* (K30076) × *A. magna* (K30097)	Leal‐Bertioli et al., 2015;
		6	10.2–70.4	6	TAG 24 × GPBD 4	Kolekar et al., 2016;
		8	42.7–83.6	8	TAG 24 × GPBD 4	Pandey, Khan, et al., 2017;
	Leaf spot resistance	5	4.6–53.00	3	*A. duranensis* (K7988) × *A. stenosperma* (V10309)	Leal‐Bertioli et al., 2009;
		28	–	13	TAG 24 × GPBD 4; TG 26 × GPBD 4	Sujay et al., 2012;
		11	1.70–6.50	0	TAG 24 × GPBD 4	Khedikar et al., 2010;
		50	5.95–27.35	10	Tifrunner × GT‐C20	Wang et al., 2013;
		20	3.41–19.12	7	Zhonghua 5 × ICGV 86699	Zhou et al., 2016;
		4	14.1–44.5	4	TAG 24 × GPBD 4	Kolekar et al., 2016;
		42	3.88–16.88	12	SunOleic 97R × NC94022	Khera et al., 2016;
		31	6.26–15.55	11	Tifrunner × GT‐C20	Pandey, Wang, et al., 2017;
		3	9.00–63.10	2	TAG 24 × GPBD 4	Pandey, Khan, et al., 2017;
	RKN resistance	10	–	7	Florunner × TxAG‐6	Burow, Starr, Park, Simpson, & Paterson, 2014;
		8	5.70–43.70	6	*A. duranensis* × *A. stenosperma*	Leal‐Bertioli et al., 2016;
	TSWV resistance	2	12.90–35.80	2	SunOleic 97R × NC94022; Tifrunner × GT‐C20	Qin et al., 2012;
		24	4.40–34.92	6	Tifrunner × GT‐C20	Wang et al., 2013;
		2	10.02–22.70	1	Florida‐ EPTM “113” × Georgia Valencia	Tseng et al., 2016;
		6	4.36–29.14	4	SunOleic 97R × NC94022	Khera et al., 2016;
		11	6.74–14.41	1	Tifrunner × GT‐C20	Pandey, Wang, et al., 2017;
	Thrips resistance	3	5.86–19.43	2	Tifrunner × GT‐C20	Wang et al., 2013;
	Bruchid resistance	44	11.00–82.00	13	VG 9514 × TAG 24	Mondal et al., 2014

Notes

PVE: percentage phenotypic variance explained; GH: growth habit; MSH: main stem height; PH: plant height; DF: days to flowering; LNB: length and number of branches; PoM: percentage of maturity; HI: harvest index; SW: seed weight; FSD: fresh seed dormancy; T: transpiration (T); TE: transpiration efficiency; LA: leaf area; CI: carbon isotope discrimination ratio; CC: canopy conductance.

a Epistatic QTLs are included for some studies.

Another significant advance in groundnut genomics has been the release of the draft genome sequences of the 1.1 Gb genome size for A‐genome progenitor (*A. duranensis,* accession V14167) and 1.38 Gb for B‐genome progenitor (*A. ipaensis,* accession K30076) (Bertioli et al., 2016). In addition, the draft genome sequence of another A‐genome progenitor accession (*A. duranensis,* accession PI475845) was generated with 1.07 Gb genome size which provided greater insights into the genome architecture and genes related to important traits such as geocarpy, oil biosynthesis and allergens (Chen, Li, et al., 2016). In the case of cultivated tetraploid genotype, a high‐quality genome assembly of ‘Tifrunner’, an important US variety with good market and growth characteristics and resistance to several diseases, was released in December 2017 (https://peanutbase.org/peanut_genome). The draft genome sequences have enabled large‐scale genomewide discovery of 515,223 indels (Vishwakarma et al., 2017) and SSRs including 105,003 SSRs in the A‐genome (Chen, Li, et al., 2016), 135,529 SSRs in the A‐genome (Zhao et al., 2017), 199,957 SSRs in the B‐genome (Zhao et al., 2017), 84,383 in the A‐genome (Luo, Ren, et al., 2017) and 120,056 in the B‐genome (Luo, Ren, et al., 2017). Further, a high‐throughput genotyping platform, an Axiom_Arachis SNP array with 58K genomewide SNPs, was developed from the analysis of DNA resequencing and RNA sequencing of 41 groundnut accessions and wild diploid ancestors against the genomes of two groundnut progenitors, that is *A. duranensis* and *A. ipaensis* (Pandey, Agarwal, et al., 2017), which was used to identify signatures of selection and tetrasomic recombination in groundnut (Clevenger et al., 2017). For understanding the genetic architecture of domestication‐related traits in groundnut, specific‐locus amplified fragment sequencing (SLAF‐seq) method was employed for large‐scale identification of 17,338 high‐quality SNPs in the whole groundnut genome, and 1,429 candidate genes for eleven agronomic traits were found using genomewide association studies in 158 peanut accessions (Zhang et al., 2017).

## 4 BREEDING

### 4.1 Focus traits and breeding methods

Priority traits in groundnut breeding include high pod yield, early maturity, high shelling percentage, high oil, resistance to biotic and abiotic stresses, fresh seed dormancy, confectionery, high oleic acid and dual‐purpose types. In the USA and other developed countries, under high input production system, the breeding focus has been maximizing yield, but in recent years, improving quality and flavour, resistance to drought and diseases have become important priorities. In Asia and Africa, the focus has been increasing pod yield with enhanced resistance to biotic and abiotic constraints and high oil content. Conventional breeding approaches such as introduction, selection, mutation and hybridization (pedigree, backcross and single‐seed descent, etc.) have been used to develop improved varieties. In the USA, although it was used extensively in the late 1950s to early 1970s, mutation breeding is little used in the present day (Holbrook & Stalker, 2003). In India, mutation breeding is still being used at Bhabha Atomic Research Center (BARC) (Mondal, Badigannavar, Kale, & Murty, 2007).

Genetic resources conserved in gene banks have been important and harbour huge potential for utilization in breeding programmes as sources of variability. Besides, recent advances in genomics have enabled integrating molecular marker‐assisted breeding approaches for selected traits, and they hold significant promise for many other traits to enhance the breeding efficiency and increase the rate of genetic gain. Brief highlights of groundnut genetic resources, breeding for specific traits and marker‐assisted breeding are provided below. Over the years, several advanced breeding and germplasm lines have been identified and developed for drought, leaf spots, rust, rosette, aflatoxin, rust and quality traits (Table 3). Genotype × environment interaction is widely reported for pod yield and other quantitative traits in groundnut (e.g., Bucheyeki, Shenkalwa, Mapunda, & Matata, 2008; Janila, Manohar, Patne, Variath, & Nigam, 2016; Jogloy, Vorasoot, Akkasaeng, Kesmala, & Patanothai, 2009; Makinde, Ariyo, & Akinbowale, 2013). Hence, multilocation and multiseason testing are required to release improved varieties. Farmer participatory variety selection (Ntare et al., 2007) has been an important approach recently in groundnut varieties’ release processes, particularly in South Asia (SA) and sub‐Saharan Africa (SSA), to better understand farmers’ trait preferences for varieties and increase farmers’ exposure to new groundnut varieties such that breeding programmes were able to better target varieties to both the ecological and market needs. Table 4 shows some of the released varieties in SA and SSA between 2000 and 2016 for their high yield and other traits including short duration, drought tolerance, rosette resistance and foliar disease resistance.

**Table 3:** Some sources of variability identified/developed for traits of breeding interest in groundnut

Trait	Source	Reference
Drought	55‐437, 55‐33, TS 32‐1, SRV 1‐3, SR 1‐96, ICG 3086, ICG 3141, ICG 2738, ICG 1163, ICG 862, ICG 8285, ICG 11855, ICG 118, ICG 2106, ICG 5827, ICG 11219, ICG 5236, ICG 6654, ICGV 91151, ICGV 94127, ICGV 92209, ICGV 91109, ICG 2213, ICG 76, ICGV 90226, ICGV 91074, ICGV 91185, ICGV 91192, ICGV 92004, ICGV 92022, ICGV 92023, ICGV 92028, ICGV 92029, ICGV 92033, ICGV 86124, ICGV‐SM 87003, ICGV 92097, ICGV 92098, ICGV 91114, ICGV 00350, ICGV 00351, ICGV 87846, ICG 14390, ICG 14778, ICGV‐SM 00537, ICGV‐SM 03535, ICGV 01263, ICGV 96155, ICGV 02266, ICGV 97183, ICGV 97182, ICGV 01232, ICGV 02189, ICG 11, ICG 37, ICG 44, ICG (FDRS) 10, ICGV 86021	Mayeux et al., 2003; Upadhyaya, 2005; Nigam et al., 2005; Hamidou et al., 2012; Monyo & Varshney, 2016;
Leaf spots	ICG 2716, ICG 3527, ICG 4747, ICG 6340, ICG 7013, ICG 7881, ICG 7884, ICG 7885, ICG 7887, ICG 7897, ICG 8123, ICG 8138, ICG 8133, ICG 8126, ICG 4983, ICG 8129, ICG 8130, ICG 8131, ICG 8922, ICG 8149	Subrahmanyam et al., 1985;
	ICG 9037, ICG 5663, ICG 721, ICG 5745, ICG 8285, ICG 6022, ICG 405, ICG 14466, ICG 6057, ICG 9449, ICG 12509, ICG 6703, ICG 10036, ICG 10384, ICG 11219, ICG 4156, ICG 9905, ICG (FDRS) 4, ICG 7878, ICGV 07210, ICGV‐SM 93531, ICGV‐SM 95714, ICGV‐IS 96802, ICGV‐IS 96808, ICGV‐IS 96827, ICGV 13192, ICGV 13193, ICGV 13200, ICGV 13206, ICGV 13228, ICGV 13229	Izge et al., 2007; GCP 2011, Kanyika et al., 2015; Monyo & Varshney, 2016; Janila, Pandey, Manohar, et al., 2016;
Rust	ICG(FDRS) 11, ICG(FDRS) 21, ICG(FDRS) 10, ICG(FDRS) 22, ICG(FDRS) 27	Reddy et al., 1987;
	ICG 1697, ICG 2716, ICG 4746, ICG 7296, ICG 7893, ICG 7899, ICG 02446, ICG 11426, 92R/70‐4, ICGV 86699, ICGV 87354, ICGV 92267, ICGV 99005, ICGV 02194, ICGV 01276, ICGV 94114, ICGV‐SM 86021, ICGV‐SM 02536, ICGV 02194, ICGV 01276, ICGV 02286, ICGV 13192, ICGV 13193, ICGV 13200, ICGV 13206, ICGV 13228, ICGV 13229, ICGV 00064, ICGV 86855	Subrahmanyam et al., 1995; Reddy et al., 2001; GCP 2011, Varshney et al., 2014; Monyo & Varshney, 2016;
Rosette	69‐101, RMP 12 and RMP 91, KH 149 A, KH 241 C, KH 241 D, QH 243 C	Ntare et al., 2002;
	24, 25, 26, ICGV‐IS 96808, ICGV‐IS 96814, ICGV‐IS 96855, ICGV‐IS 96891, ICGV‐IS 96894, UGA 2, M572.801, ICG 14705, ICG 13099, ICG 9449, ICG 15405	Mayeux et al., 2003; GCP, 2011, Monyo & Varshney, 2016;
Aflatoxin	73‐33, ICGV 89063, ICGV 89112	Mayeux et al., 2003;
	ICGV 88145, ICGV 89104, ICGV 91278, ICGV 91283, ICGV 91284	Nigam et al., 2009;
	ICG 13603, ICG 1415, ICG 14630, ICG 3584, ICG 5195, ICG 6703, ICG 6888	Waliyar et al., 2016;
Quality traits	*High oil*‐ICGV 03042, ICGV 03043, ICGV 03057, ICGV 03128, ICGV 05155, ICGV 06146, ICGV 06420, ICGV 07220, ICGV 07018, ICGV 07222	Janila, Manohar, Patne, et al., 2016; Janila unpublished
	*High oleic acid*‐ICGV 15008, ICGV 15017, ICGV 15023, ICGV 15025, ICGV 15033, ICGV 15044, ICGV 15046, ICGV 15051, ICGV 15052, ICGV 15059, ICGV 15060, ICGV 15034, ICGV 15035, ICGV 15039, ICGV 15055, ICGV 16001, ICGV 16002, ICGV 16010, ICGV 15038, ICGV 15064, ICGV 15065, ICGV 15070, ICGV 15074, ICGV 15083, ICGV 15090, ICGV 15076, ICGV 15080 ICGV 15112, ICGV 15073, ICGV 16011, ICGV 16012, ICGV 15094, ICGV 15001, ICGV 15002, ICGV 15003, ICGV 15004, ICGV 15005, ICGV 15006	Janila, Pandey, Shasidhar, et al., 2016; Janila unpublished
	*Confectionery‐*Fleur 11, GH 119‐20, H 75‐1078‐936, 47‐10, ICGV 97041, ICGV 97047, ICGV 97049, ICGV 97065, ICGV 11310, ICGV 11326, ICGV 11327, ICGV 11330, ICGV 11354, ICGV 12219, ICGV 12233, ICGV 12224, CG7, Chalimbana	Mayeux et al., 2003; Monyo & Varshney, 2016

**Table 4:** Improved groundnut varieties released between 2000 and 2016 in SA and SSA

Region	Country	# of varieties	Release name of varieties^b^	Breeding programme name of varieties	Year of release
SA	Bangladesh	2	BARI Badam‐5, BARI Badam‐6	ICGS(E) 55 [ICGV 86072], M‐5	1997
		1	ICGV 89259	ICGV 89259	2004
		1	Barichinabadam ‐ 8	ICGV 94322	2006
		2	BARI Chinabadam‐9, ICGV 96346	ICGV 96342, ICGV 96346	2010
	India	2	SG 99, Pratap Mungphali ‐ 1	ICGV 89280, ICGV 92035	2004
		1	Pratap Mungphali‐2	ICGV 92195	2005
		2	Devi, AK 303	ICGV 91114, AK 303	2006
		8	ICGV 00348, Mallika, ICR 48, VL Mungphali 1, ICGV 91114, Vijetha, Ajeya, Avtar	ICGV 00348, ICGV 00440, ICGV 07356, ICGV 86590, ICGV 91114, ICGV 93260, ICGV 93261, ICGV 93468	2008
		2	ICGV 00350, Co6	ICGV 00350, ICGV 87846	2010
		3	ICGV 00298, ALG 06‐320, ICGV 99195	ICGV 00298, ICGV 94118, ICGV 99195	2011
		1	CTMG 6	ICGV 05049	2012
		1	Co7	ICGV 00351	2013
		2	KDG 123, Phule Warna	KDG 123, KDG 128	2014
SSA	Ghana	2	Gusie‐Balin, Kpanieli	ICGV 92099, ICGV 90084	2005
		4	Oboolo, Obooshi, Otuhia, Yenyawoso	ICGV 97049, ICGV 98412, ICGV‐SM 88709, ICGV‐SM 87057	2012
		2	ICGV 86065 (ICGS(E) 34), Sameke	ICGV 86065 (ICGS(E) 34), JL 24 (ICG 7827)	2000
		3	Waliyartiga, ICG (FDRS) 4, ICG (FDRS) 10	ICG 7878, ICG (FDRS) 4, ICG (FDRS) 10	2003
	Mali	5	Nieta Tiga, Diakandapé, Baroueli, Bagui‐tana, Nisonja	ICGV 86124, Diakandapé, Baroueli, ICGV‐IS 96802, ICGV‐IS 92525	2007
		2	ICGV 86024, Yiriwa Tiga	ICGV 86024, ICGV 86015	2011
		2	ICIAR 19BT, J11	ICIAR 19BT, J11	2015
	Malawi	2	Kakoma, Nsinjiro	JL 24 (ICG 7827), ICGV‐SM 90704	2000
		1	Baka	ICG 12991	2001
		2	Chitala, Chalimbana 2005	ICGV‐SM 99568, CML851/7	2005
		7	CG8, CG9, CG10, CG11, CG12, CG13, CG14	ICGV‐SM 08501, ICGV‐SM 8503, ICGV‐SM 01731, ICGV‐SM 01724, ICGV‐SM 01514, ICGV‐SM 99551, ICGV‐SM 99556	2014
	Mozambique	2	Mametil, Mamane	ICG 12991, ICGV‐SM 90704	2002
		1	Nyanda	ICGV 93437	2004
		6	ICGV‐SM 99541, ICGV‐SM 99568, ICGV‐SM 01513, ICGV‐SM 01514, CG 7, JL 24	ICGV‐SM 99541, ICGV‐SM 99568, ICGV‐SM 01513, ICGV‐SM 01514, ICGV‐SM 83708, JL 24 (ICG 7827)	2011
	Niger	4	ICGV 86015, ICGV‐SM 85045, ICGV 87003, ICGV 87281	ICGV 86015, ICGV‐SM 85045, ICGV 87003, ICGV 87281,	2006
		5	ICG 9346, RRB, Fleur 11, J11, JL 24	ICG 9346, RRB, Fleur 11, J11, JL 24 (ICG 7827)	2010
	Nigeria	1	Samnut 24	ICIAR 19BT	2011
		2	Samnut 25, Samnut 26	ICGX‐SM‐00020/5/P10, ICGX‐SM‐00018/5/P15/P2	2013
	Senegal	6	55‐33, 78‐936, SRV1‐19, 73‐9‐11, H75‐0, PC79‐79	55‐33, 78‐936, SRV1‐19, 73‐9‐11, H75‐0, PC79‐79	2010
		8	ISAR02‐16, ISAR03‐16, ISAR05‐16, ISAR06‐16, ISAR07‐16, ISAR08‐16, ISAR09‐16, ISAR10‐16	12CS_031, 12CS_037, 12CS_069, L27, ICGV 86124, ICGV 96808, ICG 7878	2016
	South Africa	1	JL 24	JL 24 (ICG 7827)	2002
		2	ICGV 93437, ICGV‐SM 99537	ICGV 93437, ICGV‐SM 99537	2004
		1	ICGV 98369	ICGV 98369	2007
	Tanzania	2	Pendo, Sahwia	ICGMS 33, ICGMS 44	2002
		5	Nachingwea 09, Masasi 09, Mnanje 09, Mangaka 09, Naliendele 09	ICGV‐SM‐01711, ICGV‐SM‐01721, ICGV‐SM‐83708, ICGV‐SM‐99557, ICGV‐SM‐99555	2009
		3	Narinuts 2015, Kuchele 2015, Nachi 2015	ICGV ‐SM 01731, ICG 8326, ICGV‐ SM 90704	2016
	Uganda	1	Serenut 3R	ICGV‐SM 93530	2001
		2	Serenut 4R, Serenut 2R	ICG 12991, ICGV‐SM 90704	2002
		2	Serenut 5R, Serenut 6T	ICGV‐SM 93535, ICGV‐SM 99566	2010
		8^a^	Serenut 7T, Serenut 8R, Serenut 9T, Serenut 10R, Serenut 11T, Serenut 12R, Serenut 13T, Serenut 14R	SGV 99018, SGV 99019, SGV 99044, SGV 99024, SGV 99031, SGV 99048, SGV 99052, SGV 99064	2011
	Zambia	3	Nyanda, Msandile, Chishango	ICGV 93437, ICG 12991, ICGV‐SM 90704	2004
		1	MGV 5	ICGV‐SM 92741	2008
		5	MGV 6, MGV 7, Wazitatu, Wamusanga, Lupande	ICGV‐SM‐06729, ICGV‐SM‐08503, ICGV‐SM‐05534, ICGV‐SM‐03517, ICGV‐SM‐08513	2015
	Zimbabwe		ICGV 94297	ICGV 94297	2005

Note

^a^These are derivatives of a cross involving ICGV‐SM 83708 (CG7) and ICGV‐SM 90704 (Monyo & Varshney,^2016^). ^b^The varieties were released based on their performance for one or more of important traits including high yield, drought resistance, foliar disease resistance, rosette resistance, etc.

### 4.2 Genetic resources

Genetic resources are important sources of variability for traits of breeding interest and serve as reservoirs of many useful genes for the present and future groundnut improvement programmes. Several groundnut accessions are conserved globally in national and international gene banks including ICRISAT, the USA, Brazil, India and China (Ntare, Waliyar, Mayeux, & Bissala, 2006; Pandey et al., 2012). Majority of these accessions have been characterized for various morphoagronomic and biochemical traits using groundnut descriptors (IBPGR and ICRISAT 1992, Jiang & Duan, 2006; Pittman, 1995) where large variation for qualitative and quantitative traits, seed quality traits and resistance to biotic and abiotic stresses was observed (Barkley, Upadhyaya, Liao, & Holbrook, 2016). Diversity studies using molecular markers revealed generally low diversity within the cultivated types (e.g., Halward, Stalker, Larue, & Kochert, 1991; He & Prakash, 1997; Herselman, 2003; Hopkins et al., 1999; Moretzsohn et al., 2004), but moderate‐to‐high polymorphisms were also reported (e.g., Cuc et al., 2008; Mace, Phong, Upadhyaya, Chandra, & Crouch, 2006; Mace et al., 2007; Oteng‐Frimpong, Sriswathi, Ntare, & Dakora, 2015; Roomi et al., 2014).

The use of the accessions from gene banks for crop improvement is less which is attributed to the use of working collections, consisting mostly of elite breeding lines and some improved trait‐specific lines (Gowda, Upadhyaya, Sharma, Varshney, & Dwivedi, 2013). It is also costly to screen large collections for specific traits of breeding interest (Holbrook & Stalker, 2003). A subset that represents the genetic diversity facilitates easier access to the genetic resources and enhances their use in crop improvement programmes was required. Hence, core and minicore collections were established in China (Jiang et al., 2008) and USA (Holbrook, Anderson, & Pittman, 1993; Holbrook & Dong, 2005), which have been evaluated for various traits of breeding interest including disease resistance (Anderson, Holbrook, & Culbreath, 1996; Chamberlin, Melouk, & Payton, 2010; Damicone, Holbrook, Smith, Melouk, & Chamberlin, 2010; Jiang et al., 2008; Wang et al., 2011). Similarly, ICRISAT has established a core collection of 1,704 accessions (Upadhyaya, Ortiz, Bramel, & Singh, 2003) and a minicore collection of 184 groundnut accessions (Upadhyaya, Bramel, Ortiz, & Singh, 2002). Besides, a global composite collection consisting of 1,000 accessions was developed, which was further characterized using 21 SSR markers to form a reference set consisting of 300 genetically most diverse accessions (Upadhyaya, Bhattacharjee, et al., 2006). The reference set, core and minicore collections were evaluated and characterized for various traits including drought and disease resistance for use in breeding programmes (Hamidou, Rathore, Waliyar, & Vadez, 2014; Hamidou et al., 2012; Upadhyaya, 2005; Upadhyaya, Dronavalli, Singh, & Dwivedi, 2012; Upadhyaya, MallikarjunaSwamy, Goudar, Kullaiswamy, & Singh, 2005; Upadhyaya, Mukri, Nadaf, & Singh, 2012; Upadhyaya, Reddy, Gowda, & Singh, 2006; Upadhyaya, Dwivedi, Vadez, et al., 2014; Waliyar et al., 2016) and also used for association mapping (Pandey, Upadhyaya, et al., 2014).

In addition to accessions of the cultivated groundnut, gene banks hold several wild accessions. Cultivated groundnut, being originated from single‐event hybridization of diploid wild ancestors and a subsequent lack of allele exchange with the wild species due to cross‐incompatibility, has a narrow genetic base with limited variability for some traits, particularly biotic stresses. On the other hand, wild *Arachis* species are reported to harbour high levels of resistance/tolerance to multiple stresses (Foncéka, Tossim, Rivallan, Vignes, Faye, et al., 2012; Mallikarjuna, Senthilvel, & Hoisington, 2011; Simpson, Burow, Paterson, Starr, & Church, 2003; Simpson & Starr, 2001; Stalker, Tallury, Ozias‐Akins, Bertioli, & Leal‐Bertioli, 2013; Upadhyaya, Dwivedi, Sharma, et al., 2014) and also offer important variability for agronomic traits including yield (Upadhyaya, Dwivedi, Sharma, et al., 2014). Hence, several lines have been developed through interspecific hybridization to increase the variability for important traits, and some improved varieties were released. Besides, amphiploids and autotetraploids (Mallikarjuna et al., 2011), targeting‐induced local lesions in genomes (TILLING) populations (Knoll et al., 2011), multiparent advanced generation intercross (MAGIC) populations (Janila, Variath, et al., 2016) and chromosome segment substitution (CSSL) lines (Foncéka, Tossim, Rivallan, Vignes, Lacut, et al., 2012) have been developed and form important resources of groundnut breeding.

### 4.3 Breeding for specific traits

#### 4.3.1 Drought

With more than 70% of groundnut area being in the semiarid tropics (Pandey, Guo, et al., 2014), drought is a major production constraint. Early season, midseason and end‐of‐season drought are important forms of drought, but end‐of‐season (terminal) drought that affects the seed development is more critical (Nigam, Nageswara Rao, & Wright, 2002; Williams, Rao, & Rao, 1985). Over the years, a large number of accessions and lines have been identified as sources of drought resistance (Hamidou et al., 2012; Mayeux, Waliyar, & Ntare, 2003; Monyo & Varshney, 2016; Nigam et al., 2005; Upadhyaya, 2005). Breeding for drought exploits both early maturity and drought resistance/tolerance mechanisms to develop improved varieties. Early maturity enables escape from drought stress conditions (Janila, Nigam, Pandey, Nagesh, & Varshney, 2013; Williams et al., 1985), while resistance/tolerance is usually attributed to water use efficiency, root depth and/or water extraction capacity for high yield. Empirical approach or trait‐based approach or a combination of both is used for phenotyping for drought resistance (Janila & Nigam, 2013). The empirical approach involves selection based on pod and grain yield under imposed drought stress conditions. The trait‐based approach involves phenotyping for traits such as HI, total amount of water transpired (T), TE and water use efficiency (WUE). Positive correlations were reported between TE and pod yield under water‐stressed environments (Devi et al., 2011; Sanogo, 2016). Because WUE and TE are difficult to measure routinely, surrogate traits such as SLA and SCMR are used. Significant correlations have been reported between TE and surrogate traits (Devi et al., 2011; Nageswara Rao et al., 2001).

The evidences about the usefulness of surrogate traits, however, are not consistent in that high SCMR and low SLA may not always lead to higher pod yield. For example, the preponderance of nonadditive effects and poor relationship between surrogate traits and pod yield were observed (Hamidou et al., 2012; Krishnamurthy et al., 2007; Sanogo, 2016). Janila, Manohar, Rathore, and Nigam (2015) observed low heritability for SCMR and SLA. On the other hand, high correlations of both SCMR and SLA with pod yield and other economic traits such as 100‐seed weight were reported (Janila et al., 2015; Songsri et al., 2009; Upadhyaya, 2005; Upadhyaya et al., 2011). High heritability and a lower G × E interaction for the surrogate traits were also reported (Songsri et al., 2009; Upadhyaya et al., 2011). Varshney et al. (2009) reported moderate‐to‐high heritability for drought‐related traits with alleles having moderate additive effects identified. Additive and both additive and nonadditive effects were also reported (Lal, Hariprasanna, Rathnakumar, Gor, & Chikani, 2006; Nigam et al., 2001). A combined use of the empirical and trait‐based selection approaches has been suggested under drought stress conditions (Devi et al., 2011; Janila et al., 2015; Nigam et al., 2005) as it would be advantageous in selecting genotypes which are more efficient water utilizers or partitioners of photosynthates into economic yield.

#### 4.3.2 Leaf spots

ELS and LLS are caused by *Cercospora arachidicola* Hori and *Cercosporidium personata* (Berk & Curt.). Deighton, respectively, are the most common and serious diseases of groundnut, which can cause pod yield losses of over 50% (Mayeux & Ntare, 2001; McDonald, Subrahmanyam, Gibbons, & Smith, 1985). Field and laboratory screening methods involve sowing genotypes in replicated plots with rows of a highly susceptible cultivar arranged systematically throughout the trial with good disease development ensured through the provision of inoculum (McDonald et al., 1985). A 9‐point disease scale is used for measuring reactions separately for the two leaf spots. Earlier germplasm screenings resulted in the identification of promising lines for resistance sources (Subrahmanyam, Moss, McDonald, Subba Rao, & Rao, 1985), and since then, many additional lines have become available as good sources of resistance (GCP 2011; Izge, Mohammed, & Goni, 2007, Janila, Pandey, Manohar, et al., 2016; Kanyika et al., 2015; Monyo & Varshney, 2016).

#### 4.3.3 Rust

Groundnut rust, caused by *Puccinia arachidis* Speg., is an economically important disease that significantly reduces the pod and fodder yield and oil quality. Protocols for screening genotypes at field condition involve the use of infector row technique (Subrahmanyam et al., 1995). Reviews on groundnut breeding for rust resistance are available (Mondal & Badigannavar, 2015; Subrahmanyam et al., 1997; Wynne, Beute, & Nigam, 1991). Earlier rust screening efforts identified some advanced rust‐resistant lines such as ICG (FDRS) series (Reddy, Nigam, Dwivedi, & Gibbons, 1987). Later, more accessions and advanced lines were identified (GCP 2011; Monyo & Varshney, 2016; Reddy, Nigam, Rao, & Reddy, 2001, Subrahmanyam et al., 1995; Varshney et al., 2014). Some of these lines combine rust and leaf spot resistance.

#### 4.3.4 Rosette

Groundnut rosette disease (GRD) caused by the groundnut rosette virus (GRV), groundnut rosette assistor virus (GRAV) and satellite RNA (Janila & Nigam, 2013; Reddy, Nigam, & Reddy, 1995) is a devastating disease. A method for simultaneous detection of the three causal agents has been published (Anitha, Monyo, & Okori, 2014). Sources of resistance were first discovered in cultivars from Burkina Faso and Cote d'Ivoire in 1952 (Ntare, Olorunju, & Hildebrand, 2002; Subrahmanyam, Hildebrand, Naidu, Reddy, & Singh, 1998). Resistance among these cultivars was effective against both chlorotic and green rosette forms of the disease and was governed by two independent recessive genes (Nigam & Bock, 1990; Olorunju, Kuhn, Demski, Misari, & Ansa, 1992). Breeding through utilizing the cultivars resulted in the development of long‐duration Virginia cultivars and early and medium maturing Spanish types (GCP, 2011; Mayeux et al., 2003; Monyo & Varshney, 2016, Ntare et al., 2002).

#### 4.3.5 Aflatoxin

Aflatoxin contamination induced by *Aspergillus flavus* and *A. parasiticus* is a major constraint to the global trade of groundnut. Low‐altitude warmer ecologies with low precipitation support high occurrence and distribution of *Aflatoxigenic Aspergilli* in soil and high aflatoxin B1 contamination in groundnut (Monyo et al., 2012). Three resistance mechanisms have been focuses of aflatoxin resistance breeding: (a) preharvest natural seed infection, (b) aflatoxin production and (c) in vitro seed colonization (IVSC). Nigam et al. (2009) described a large number of groundnut lines that showed IVSC resistance (15% or fewer seeds colonized) and seed infection resistance (<2% seed infection) including five elite lines recommended for cultivation in SA. In WCA, three varieties were reported for resistance to aflatoxin (Mayeux et al., 2003). More recently, seven accessions with consistent very low aflatoxin accumulation were identified (Waliyar et al., 2016). However, G × E interaction remains a major issue in screening for aflatoxin resistance (Nigam et al., 2009), and generally, little progress has been made in using conventional breeding for enhancing host–plant resistance to aflatoxin contamination (Waliyar et al., 2016). Even if some elite lines were recommended for cultivation in India (Nigam et al., 2009), so far no prominent variety has been officially released with aflatoxin resistance. Two varieties (J 11 and 55‐437) released for yield and agronomic performance in WCA are known to have a good level of resistance and serve as standard checks. Recent efforts using biotechnology options have reported a high level of resistance in groundnut by overexpressing antifungal plant defensins MsDef1 and MtDef4.2 and through host‐induced gene silencing of aflM and aflP genes from the aflatoxin biosynthetic pathway (Sharma et al., 2018).

#### 4.3.6 Quality

Oil and oleic acid content and confectionery traits are among the important quality traits. Various physical sensory, chemical and nutritional factors determine the quality of groundnut for which substantial genetic variability exists (Dwivedi & Nigam, 2005). Near‐infrared reflectance spectroscopy (NIRS), a robust and nondestructive method, is gaining popularity for the estimation of oil, protein, carbohydrate and fatty acid contents (Janila & Nigam, 2013). It is also cost‐effective compared with wet chemistry. At ICRISAT, a large number of accessions screened had 34%–55% oil content (Dwivedi & Nigam, 2005). Several advanced lines for high oil content have also been recently developed (Janila, Manohar, et al., 2016; Janila unpublished). In the case of oleic acid content, very few lines are officially released, specifically for high O/L ratio (e.g., SunOleic 95R and SunOleic 97R in the USA; PC 223 K8 and PC 223 K9 in South Africa). With regard to confectionery types, large number of varieties have been identified (Mayeux et al., 2003; Monyo & Varshney, 2016).

### 4.4 Marker‐assisted breeding

Genomic tools enhance crop breeding process by increasing the efficiency and speed of precision breeding to develop improved varieties. Diagnostic molecular markers linked with traits of breeding interest (or major effect QTLs) were identified for root‐knot nematode (Choi et al., 1999; Chu, Holbrook, Timper, & Ozias‐Akins, 2007; Church, Simpson, Burow, Paterson, & Starr, 2000; Garcia, Stalker, Schroeder, & Kochert, 1996; Simpson, 2001), rust (Khedikar et al., 2010; Mondal, Badigannavar, & D'Souza, 2012), rust and LLS (Kolekar et al., 2016; Sujay et al., 2012), nutritional quality traits (Chen, Wang, Barkley, & Pittman, 2010; Chu, Holbrook, & Ozias‐Akins, 2009; Sarvamangala et al., 2011; Wilson et al., 2017), TSWV (Tseng, Tillman, Peng, & Wang, 2016) and growth habit (Li et al., 2017). Some of these linked markers have been validated and deployed for marker‐assisted selection (MAS) and marker‐assisted backcrossing (MABC). In the USA, MAS has been used for pyramiding nematode resistance and high oleic trait (Chu et al., 2011). At ICRISAT, MABC was employed to transfer a major rust resistance QTL from GPBD 4 to three popular varieties (ICGV 91114, JL 24 and TAG 24) resulting in the development of rust resistance lines with 56%–96% increase of pod yield (Varshney et al., 2014). Some of these lines were also found to be resistant to LLS with 39%–79% of higher mean pod yield (Janila, Pandey, Manohar, et al., 2016). Besides, MAS and MABC were used to enhance the oil quality traits in three groundnut varieties (ICGV 06110, ICGV 06142, and ICGV 06420) by transferring FAD2 mutant alleles from SunOleic 95R. A large number of lines with increased oleic acid in the range of 62%–83% were identified (Janila, Pandey, Shasidhar, et al., 2016), which are currently being evaluated for yield (Janila, pers. Comm.). At Dharwad University of Agricultural Sciences in India, MABC was used to improve JL 24 with GPBD 4 as donor parent (Yeri & Bhat, 2016). Similarly, MABC was employed to improve TMV 2 for LLS and rust using GPBD 4 where two backcross lines showed enhanced resistance to LLS and rust along with 71.0% and 62.7% increase of pod yield over TMV 2 (Kolekar et al., 2017). In the case of other important quantitative traits such as drought tolerance and yield components, QTL analyses using biparental populations revealed few major rather several small‐effect QTLs. Genomewide association studies for 50 agronomic traits using 300 genotypes from the “reference set” identified a total of 524 highly significant MTAs for 36 traits (Pandey, Upadhyaya, et al., 2014) indicating complex genetic control. Breeding approaches such as marker‐assisted recurrent selection and genomic selection are the preferred approaches for introgression of a larger number but small‐effect QTLs. But such approaches have not been widely used in groundnut.

## 5 CONCLUSION AND FUTURE PERSPECTIVES

Significant progress has been made in groundnut genetics, genomics and breeding, thus contributing to the increased productivity and production of groundnut globally although the rate of increase varies among regions. It is worth mentioning that the progress has been achieved through strong partnership and collaborations between scientists from national research systems, international research institutes, universities, and private research organizations and service providers. Globally, large numbers of groundnut lines were identified or developed as sources of variability for important traits and many improved varieties were released for target environments by breeding programmes. The last decade has witnessed the rapid development of genomic tools helping to better understand the groundnut genome. MAS and MABC have proved useful for selected traits. Emerging trait mapping approaches are expected to help the search for linked markers for other traits and develop diagnostic markers for breeding applications. The availability of the diploid and tetraploid genome sequences will provide more opportunities to identify the useful genetic variation for breeding at a genome scale, discover the genes of breeding interest and identify additional molecular markers amenable for high‐throughput genotyping. High‐throughput genotyping technologies are advancing fast with genotyping costs getting cheaper. It will not be far for such technologies to be routinely utilized by many breeding programmes, if not all, for screening segregating populations, purity testing, genetic mapping, targeted resequencing of specific genomic regions and other studies. In summary, groundnut improvement tools are available to exploit and build on past achievements for new discoveries to enhance and accelerate the genetic gain of breeding programmes such that processes for the development and release of improved varieties are speedy, technically efficient and cost‐effective.

## AUTHOR CONTRIBUTIONS

Haile Desmae wrote the first draft and incorporated all inputs from the coauthors and editors. Manish K Pandey, Emmanuel Monyo and Rajeev K Varshney revised the first draft and made suggestions for improving the manuscript. Pasupuleti Janila, Babu N Motagi, Patrick Okori, Omari Mponda, David Okello, Dramane Sako, Candidus Echeckwu, Richard Oteng‐Frimpong, Amos Miningou and Chris Ojiewo provided country‐specific data and information regarding groundnut improvement and accordingly revised the manuscript.

## CONFLICT OF INTEREST

The authors declare no conflict of interest.
